# A role for TRPC5 in cold sensing, finally

**DOI:** 10.1007/s00424-021-02588-1

**Published:** 2021-06-26

**Authors:** Johannes Oberwinkler

**Affiliations:** 1 Institut für Physiologie und Pathophysiologie, Philipps-Universität Marburg, 35037 Marburg, Germany; 2 Center for Mind, Brain and Behavior (CMBB), Philipps-Universität Marburg and Justus-Liebig-Universität Giessen, 35032 Marburg, Germany

Teeth are made of exceptionally hard material, mainly dentin, covered with a layer of enamel. The hollow interior of teeth is filled with soft tissue (called pulp) and is densely innervated by sensory neurons, which also protrude partly into tiny tubules formed by dentin [3]. Despite their rigid exterior, teeth are nonetheless capable of sensing environmental stimuli such as temperature changes. As many will know from first-hand experience during sensitivity testing routinely performed by dentists, cold stimulation of teeth can cause a sudden, sharp, and intense pain. This is the expected, physiological reaction of intact, healthy teeth. However, in dentin hypersensitivity, a rather common condition, innocuous cool stimuli, such as cold wind, may be sufficient to cause significant tooth pain inciting patients to seek treatment [7].

In human skin, cold sensitivity depends largely on neurons expressing TRPM8, a cold- and menthol-responsive cation channel [2, 4]. An additional contribution of TRPA1 channels, especially for the detection of strong, noxious cold stimuli has been reported repeatedly, but remains controversial [2]. There are several other ion channels sensitive to cooling, but for many of those no role in sensory signaling in vivo has been established so far [2]. A case in point is TRPC5, which forms channels robustly activated by decreasing temperatures when heterologously expressed, but for which no function in the somatosensory system has been established unequivocally until now [2, 10].

Cold sensing in teeth is even less well understood than in skin, likely reflecting the fact that the anatomical situation is highly challenging to the experimenter, with the site of cold transduction rendered rather inaccessible by the tough shield of enamel and dentin. To circumvent this problem, Bernal et al. developed a preparation to record from single nerve fibers in the nerve that innervates the teeth of the lower jaw [1]. Working with mice, this preparation enabled the genetic dissection of the responses of dental sensory neurons to cold stimuli. Interestingly, but maybe not unexpectedly, Bernal et al., in a recent study published in Science Advances [1], found that deleting TRPM8, TRPA1, and TRPC5 channels individually did not eliminate the cold response from dental sensory neurons. Surprisingly, however, they found that eliminating TRPC5 channels had the largest effect roughly halving the number of cold-sensitive fibers. Eliminating TRPA1 channels in addition (by using TRPC5/TRPA1 double knock-out mice) further reduced the number of neurons reacting to cold stimuli, while no contribution of TRPM8 channels to the cold sensitivity of dental sensory neurons could be identified. These data confirm earlier findings that TRPM8 and TRPA1 channels are not sufficient to explain dental cold sensitivity [6], but importantly also identify TRPC5 as a relevant transduction channel.

When Bernal et al. looked (with Ca^2+^ imaging) at the responses of isolated cell bodies from dental neurons (identified by fluorescent tracers), they obtained results that were markedly different: Most cold responses from isolated neurons were dependent on TRPM8, with only minor (but robust) contributions from TRPA1 and TRPC5. The authors offer a compelling set of data to resolve this discrepancy: They identified a strong expression of TRPC5 in mouse and human odontoblasts, the cell type situated at the highly innervated pulp-dentin interface, the location best suited for sensing external temperature changes reaching the inner parts of teeth.

While much work remains to be done to fill in the details (including the direct demonstration of functional TRPC5 channels in odontoblasts), the work of Bernal et al. strongly favors a model in which odontoblasts are sensory cells excited by cold stimuli which signal their activation to neurons close by, possibly via the release of ATP [8]. The exciting findings of Bernal et al. therefore add significantly to the emerging concept that somatosensory neurons are not the only agents in stimulus detection, but work in concert with, or even secondary to other, non-neuronal cells, such as odontoblasts [1, 8], Merkel cells [5], or keratinocytes [9].
